# Supplementary material for: Quantifying societal emotional resilience to natural disasters from geo-located social media content
Source: PLoS One. 2022 Jun 16;17(6):e0269315. doi: 10.1371/journal.pone.0269315 (PMC9202846; doi:10.1371/journal.pone.0269315)
Supplement: S1 Table — The first two columns are the name, year, and location of the hurricane. The next two are the bounding box from which the geo-located tweets were sampled from. The following three date columns are the dates from which the tweets were sampled and the US Landfall respectively. In the case of Alabama, the US Landfall represents when the official announcement was made by the White House. (PDF) [file pone.0269315.s001.pdf]

Hurricane	Location	Coordinate Box (lon, lat)		Dates		Hit Date
		Corner	Opposite Corner	Start	End	
Irma (2017)	Florida	(-83.152,24.373)	(-79.324,32.155)	08-25	09-22	09-09
Harvey (2017)	Houston	(-98.0143,26.928)	(-89.826,30.846)	08-12	09-02	08-24
Florence (2017)	Carolinas	(-82.197,31.709)	(-75.193,37.080)	08-25	09-24	09-14
Dorian (2018)	Carolinas	(-80.264, 32.490)	(-76.477, 35.197)	08-24	09-10	09-06
Dorian (2019)	Florida	(-81.710, 26.932)	(-79.565, 30.920)	08-24	09-10	09-03
Dorian (2019)	Alabama	(-86.789, 29.664)	(-83.377, 31.536)	08-24	09-10	09-04
