# Supplementary material for: Quantifying societal emotional resilience to natural disasters from geo-located social media content
Source: PLoS One. 2022 Jun 16;17(6):e0269315. doi: 10.1371/journal.pone.0269315 (PMC9202846; doi:10.1371/journal.pone.0269315)
Supplement: S2 Table — We tested 4 different models in order to find the best fits. In all cases except Dorian in the Carolinas, the exponential fit had the lowest sum of square errors (SSE). (PDF) [file pone.0269315.s006.pdf]

	Models			
	Exponential	Gaussian	Lorentzian	Quadratic
Harvey	1.28	1.27	1.27	2.19
Irma	0.72	1.86	0.87	1.23
Florence	1.45	2.88	2.55	2.02
Dorian	10.38	9.58	13.17	13.93
