# Supplementary figures and images for: Quantifying societal emotional resilience to natural disasters from geo-located social media content
Source: PLoS One. 2022 Jun 16;17(6):e0269315. doi: 10.1371/journal.pone.0269315 (PMC9202846; doi:10.1371/journal.pone.0269315)

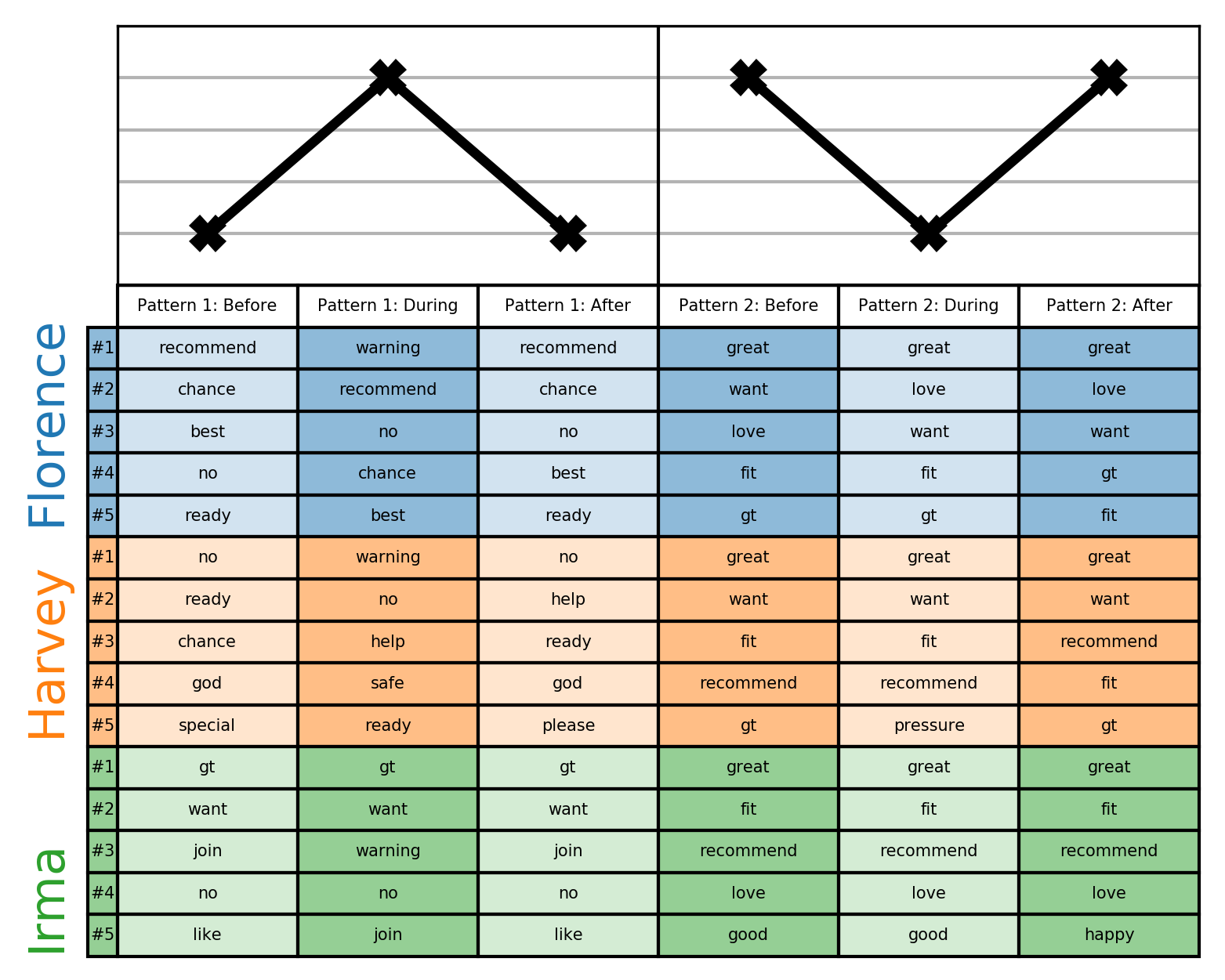

Supplement: S1 Fig — The first cluster showed more caution related words with a higher TF-IDF during the hurricane than before or after, such as “warning’, “safe”, and “help”. In contrast, the second cluster had very similar words across all time periods. The drop in TF-IDF of these similar words in the second cluster can be attributed to the increase in caution related words in the first cluster. (TIF) [file pone.0269315.s002.tif]

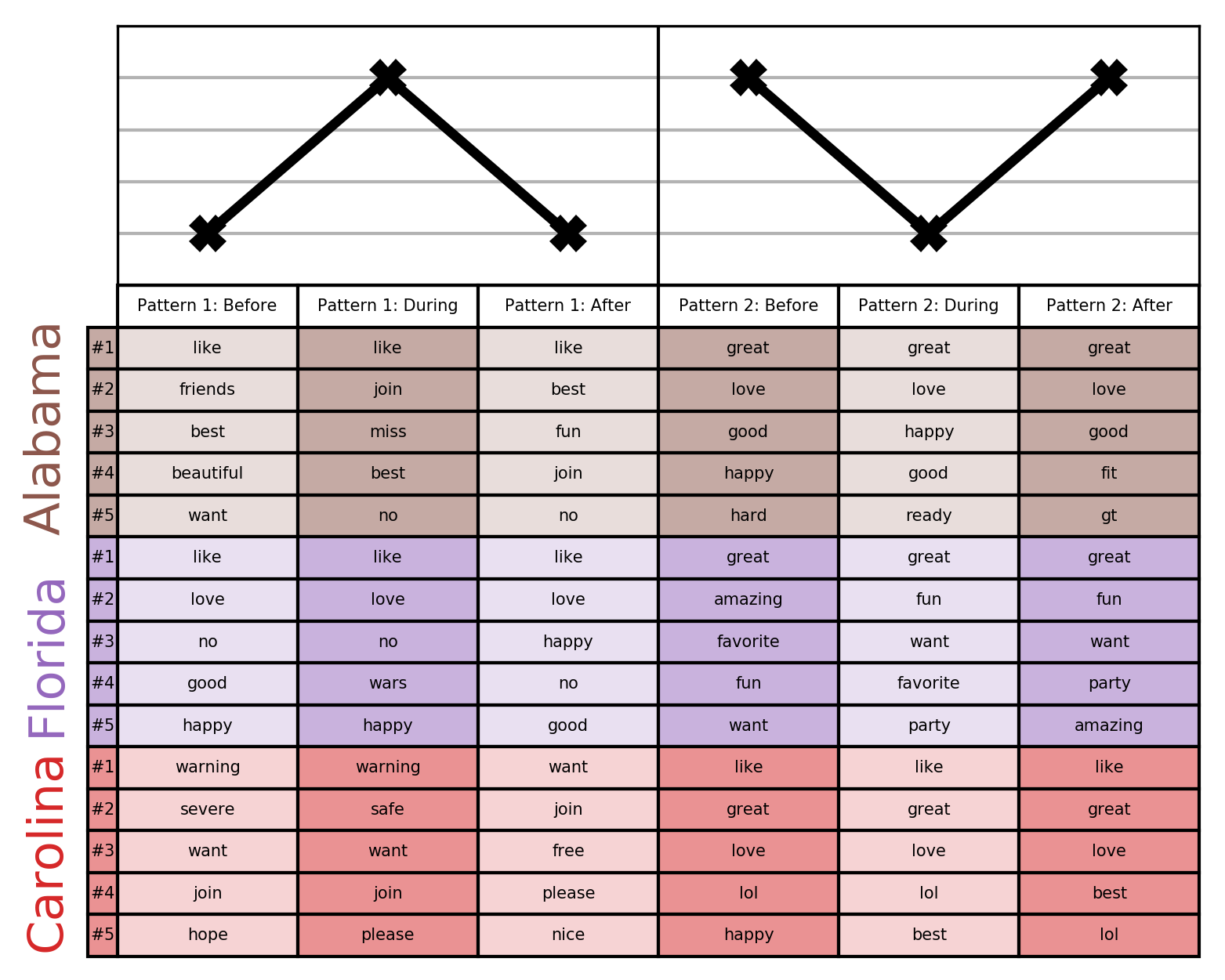

Supplement: S2 Fig — For Florida and Alabama, the words in both clusters are similar across each time period. The Carolinas, on the other hand, show an increase in caution related words throughout (seen in cluster 1) and thus a decrease in the commonly said words (seen in cluster 2). (TIF) [file pone.0269315.s003.tif]

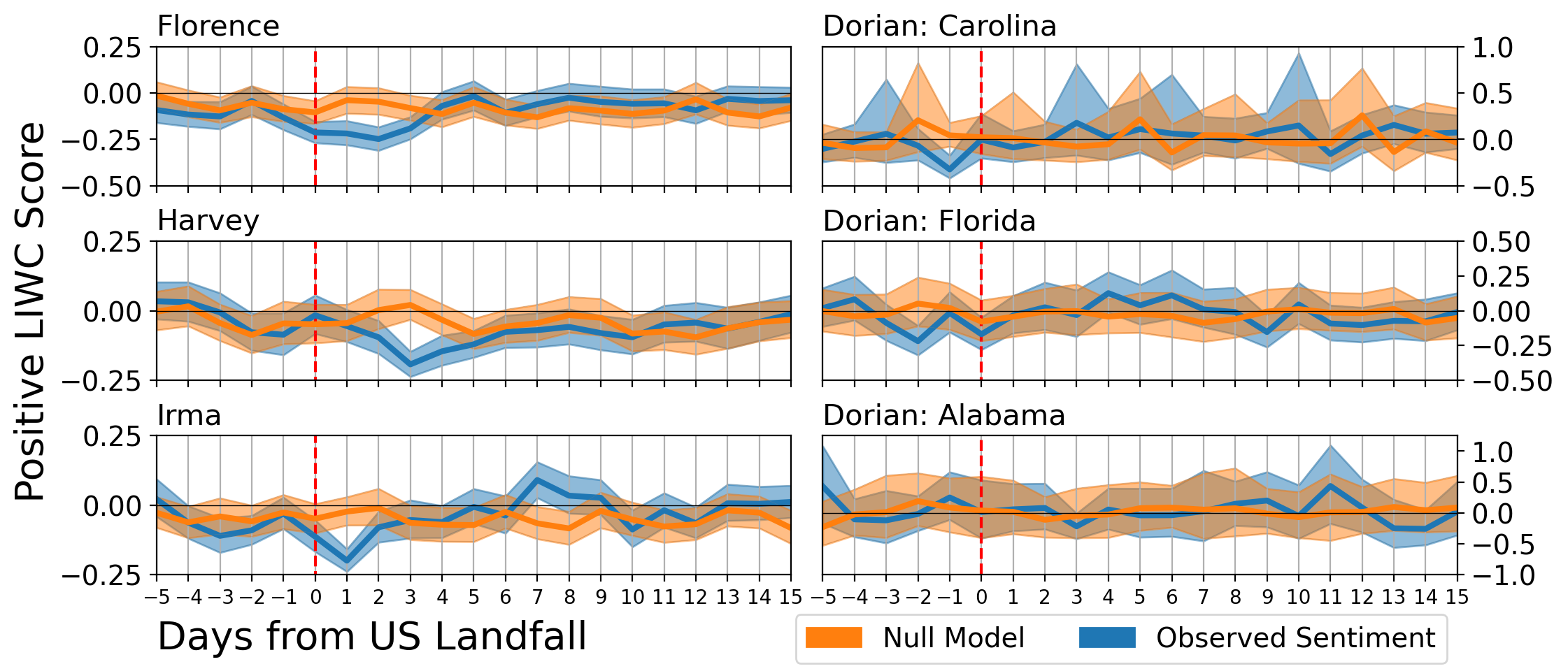

Supplement: S3 Fig — For each day, we calculated the 2.5th, 50th, and 97.5th percentile of the bootstrapped sentiments in both the original data and the null model. The bold line is the median of the observed data and the null model while the shaded areas represent the 95% confidence interval. Any day in which the confidence intervals does not intersect represents a statistically significant change in sentiment. In all locations with landfall, the positive word ratio had no significant change with the exception of Hurricane Irma 7 days after landfall. (TIF) [file pone.0269315.s004.tif]

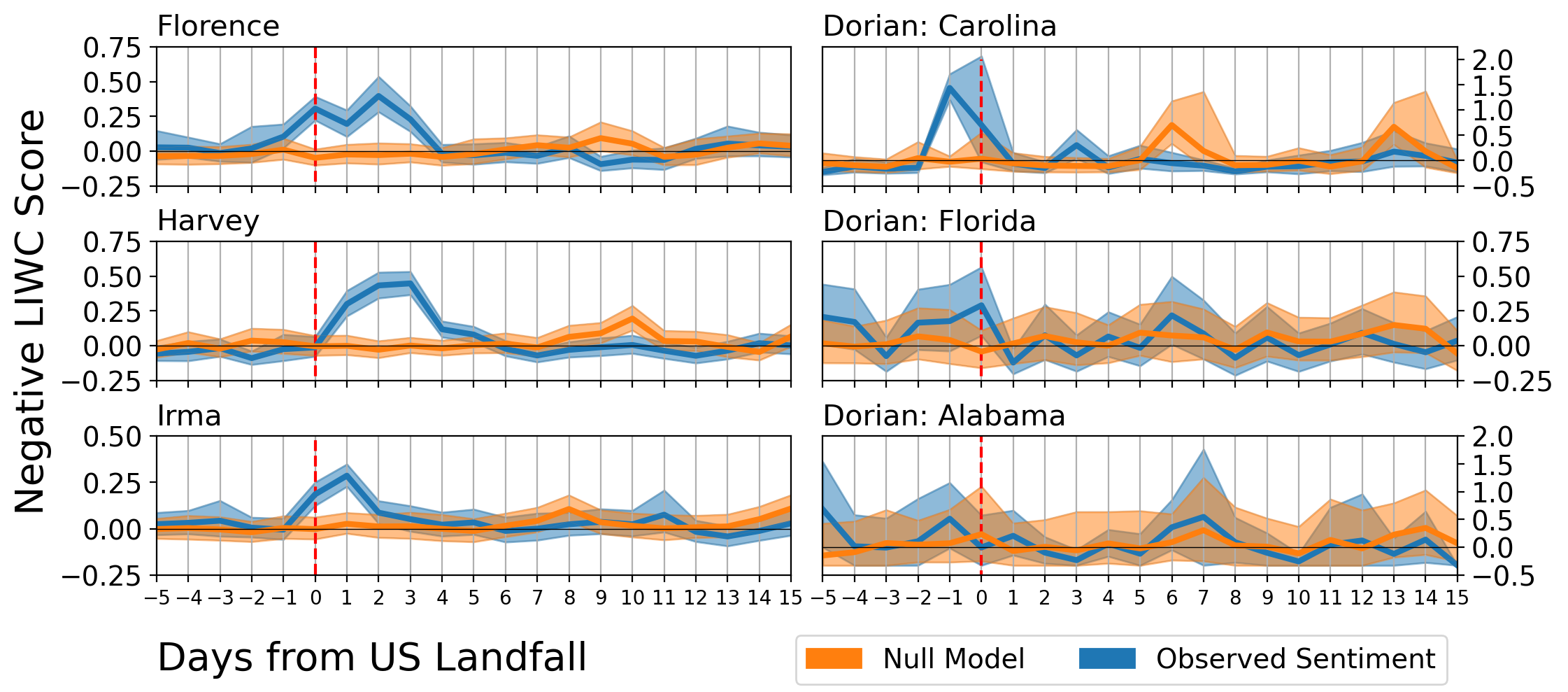

Supplement: S4 Fig — For each day, we calculated the 2.5th, 50th, and 97.5th percentile of the bootstrapped LWNR in both the original data and the null model. The bold line is the median of the observed data and the null model while the shaded areas represent the 95% confidence interval. Any day in which the confidence intervals does not intersect represents a statistically significant change in the LWNR. Hurricanes Florence, Harvey, and Irma caused a significant increase in the LWNR starting at landfall while the LWNR in Dorian (Carolinas) dropped 2 days beforehand. (TIF) [file pone.0269315.s005.tif]
